# Adult Mouse Kidney Stem Cells Orchestrate the De Novo Assembly of a Nephron via Sirt2‐Modulated Canonical Wnt/*β*‐Catenin Signaling

**DOI:** 10.1002/advs.202104034

**Published:** 2022-03-22

**Authors:** Xiaobin Han, Zhongjie Sun

**Affiliations:** ^1^ Department of Physiology University of Tennessee Health Science Center Memphis TN 38163 USA

**Keywords:** de novo generation, kidney organoids, kidney stem cells, Sirt2, Wnt signaling

## Abstract

Generation of kidney organoids using autologous kidney stem cells represents an attractive strategy for treating and potentially replacing the failing kidneys. However, whether adult mammalian kidney stem cells have regenerative capacity remains unknown. Here, previously unidentified adult kidney Sca1^+^ Oct4^+^ stem/progenitor cells are isolated. Interestingly, culturing these cells leads to generation of kidney‐like structures. First, the assembly of self‐organizing 3D kidney‐like structures is observed. These kidney organoids contain podocytes, proximal tubules, and endothelial cells that form networks of capillary loop‐like structures. Second, the differentiation of kidney stem cells into functionally mature tubules and self‐organizing kidney‐shaped structures in monolayer culture that selectively endocytoses dextran, is shown. Finally, the de novo generation of an entire self‐organizing nephron from monolayer cultures is observed. Mechanistically, it is demonstrated that Sirt2‐mediated canonical Wnt/*β*‐catenin signaling is critical for the development of kidney organoids. Thus, the first evidence is provided that the adult mouse kidney stem cells are capable of de novo generating kidney organoids.

## Graphical abstract

This study demonstrates that stem cells reside in the adult mammalian kidneys. The finding provides the first evidence that adult mouse Sca1^+^Oct4^+^ kidney stem cells are capable of de novo generating a nephron in vitro. Mechanistically, Sirt2‐mediated canonical Wnt/*β*‐catenin signaling is critical for the development of kidney organoids.

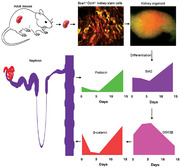

## 1 Introduction

Over the past decade, several studies have reported the presence of stem cells in the adult mammalian kidney.^[^ ^1^, ^2^, ^3^, ^4^, ^5^, ^6^ ^]^ To do so, a single (Sca1, CD45, or CD133) or combination of double stem cell markers (CD24 and CD133) were often used to isolate adult kidney stem/progenitor cells from mammals including humans.^[^ ^2^, ^7^, ^8^, ^9^, ^10^, ^11^, ^12^ ^]^ Nevertheless, there have been no successful attempts for generating kidney organoids from these cells. Therefore, whether there exist adult kidney stem cells remains widely debated.^[^ ^13^ ^]^ One of the major obstacles to isolate adult kidney stem cells, if there is any, is lack of specific marker for kidney stem cells. Therefore, a more comprehensive strategy may be required to identify adult kidney stem cells by using a combination of specific markers of embryonic stem cells and renal progenitors. Vigneau et al. reported that mouse embryonic stem cell‐derived embryoid bodies expressed Oct4 and CD133 stem cell markers, and it generated progenitors expressing renal markers cadherin‐11, WT‐1, Pax‐2, and Wnt4. Notably, these progenitors can integrate into renal proximal tubules in vivo.^[^ ^14^ ^]^

Here we developed a methodology for isolation of kidney stem cells from adult mouse kidneys by using Sca1 and Oct4 stem cell markers. Interestingly, these Sca1^+^ Oct4^+^ cells also expressed stem cell markers CD133, CD34, CD45, and renal markers cadherin‐11, WT‐1, Pax‐2, and Wnt4. Sca1^+^ Oct4^+^ kidney stem cells can form self‐organizing kidney organoids in culture. Thus far Oct4 has not been used for successful isolation of kidney stem cells that can generate kidney organoids. SIRT2, an NAD‐dependent deacetylase, plays an important role in cell proliferation, differentiation, and metabolism.^[^ ^15^ ^]^ The Wnt signaling is also critical to cell proliferation and differentiation.^[^ ^16^ ^]^ Sirt2 and Wnt signaling are involved in the embryonic organ development. We found that expression of Sirt2 was upregulated, which modulated the dynamics of canonical Wnt/*β*‐catenin signaling during kidney organoid development. Notably, these kidney organoids developed glomerular structure with a de novo vascular network devoted with podocytes. Furthermore, differentiation of kidney stem cells in monolayer cultures revealed a single lobe kidney‐like structure with the function of selective uptake of dextran in vitro. Finally, we demonstrated a de novo generation of an entire nephron from differentiated monolayer cultures of kidney stem cells. The efficiency of these Sca1^+^ Oct4^+^ kidney stem cells to form self‐organizing kidney organoids, together with their high levels of maturity and functionality, provide potential window of opportunities for studying mammalian kidney development and personalized kidney regeneration.

## 2 Results

### 2.1 Isolation of Sca1^+^ Oct4^+^ Cells Resided in the Adult Mouse Kidney

**Figure 1:**
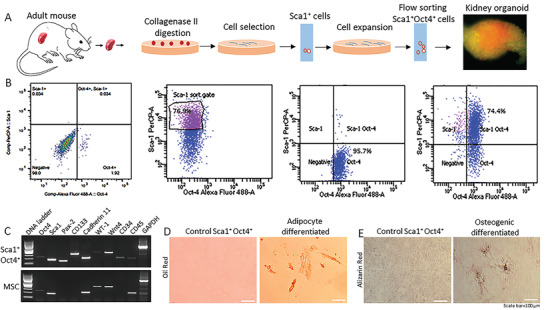
The isolated Sca1^+^ Oct4^+^ cells form self‐organizing organoids. A) Working flow for isolation of Sca1^+^ Oct4^+^ cells. B) Purification of Sca1^+^ cells by flow cytometry and confirmation of Sca1^+^ Oct4^+^ expression by double antibody labeling. C) RT‐PCR analysis of stem cell and renal markers in mouse mesenchymal stromal cells (MSCs) versus the isolated Sca1^+^ Oct4^+^ cells. D) Adipocyte differentiation of Sca1^+^ Oct4^+^ cells. Scale bar, 100 µm. E) Osteogenic differentiation of Sca1^+^ Oct4^+^ cells. Scale bar, 100 µm. Data are representative of three independent experiments.

To isolate stem cells that reside in the kidney, we adapted a strategy to culture single cells isolated from collagenase II‐digested adult mouse kidneys (**Figure** **1**A); these kidneys were not previously subjected to acute or chronic injury.^[^ ^17^ ^]^ Amongst the isolated cells, we selected for stem/progenitor cells with a 24‐hour culture in serum‐free Medium A (DMEM/F12, 0% FBS and 1% penicillin streptomycin), followed by a ≤ 7‐day culture in Medium B (DMEM/F12, 10% FBS and 1% penicillin streptomycin). Cells that survived selection were cultured in Medium C (DMEM/F12, 20% FBS, 1% penicillin streptomycin, 20 ng mL^−1^ stem cell factor, and 25 ng mL^−1^ bFGF) for up to 6 weeks to promote stem/progenitor cell recovery. From these cultures, we isolated stem cell antigen‐1 positive (Sca1^+^) cells (Figure 1A) with a mouse Sca1^+^ selection kit. These Sca1^+^ cells were further purified by flow cytometry, and ≈97% of sorted Sca1^+^ cells were confirmed Sca1^+^ Oct4^+^ by double labeling with Sca1 and Oct4 antibodies (Figure 1B). Moreover, gene expression analyses indicated that these Sca1^+^ Oct4^+^ cells also expressed hematopoietic stem cell markers CD133, CD45, and CD34, as well as renal markers Pax‐2, WT‐1, cadherin‐11, and Wnt4.^[^ ^14^ ^]^ (Figure 1C). Their multipotency was validated by adipocyte and osteogenic differentiation studies (Figure 1D,E). To assess whether the isolated Sca1^+^ Oct4^+^ cells were kidney stem cells, we used cells to perform a series of additional studies. We first tested whether these Sca1^+^ Oct4^+^ cells could generate kidney organoids. To do so, we seeded cells (≈1×10^6^) in a regular, untreated 10 cm petri dish containing Medium C, as described above. These Sca1^+^ Oct4^+^ cells surprisingly formed self‐organizing 3D organoids overnight in culture. These Sca1^+^ Oct4^+^ cells‐derived kidney organoids further developed into larger organoids (>300 µm in diameter) over 14 days. Notably, we have cultured these Sca1^+^ Oct4^+^ cells for over 2.5 years with more than 150 passages without losing the capacity of forming kidney organoids (Figure S1A, Supporting Information), which is a golden standard for determination of kidney stem cells in the field^13^.

### 2.2 Sca1^+^ Oct4^+^ Cell‐Derived Kidney Organoid Contains Glomerular‐Like Structure with a De Novo Vascular Network

**Figure 2:**
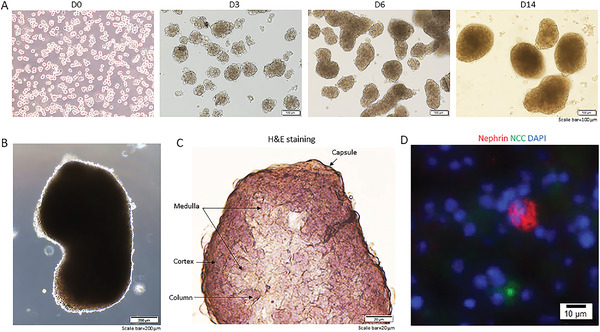
Kidney organoids derived from the isolated Sca1^+^ Oct4^+^ cells develop kidney structures and glomerular lineage. A) The time‐course of the development of Sca1^+^ Oct4^+^ cell‐derived kidney organoids in culture. Scale bar, 100 µm. B) Bright field of kidney‐shaped KSCs‐derived organoid. Scale bar, 200 µm. C) Cryosection of kidney organoids (H&E staining) revealing kidney structures including cortex, medulla, column, and capsule. Scale bar, 20 µm. D) Cryosections of kidney organoids were further processed with immunofluorescent staining. The photo shows glomerulus‐like structure with positive staining for nephrin (red) and distal tubule‐positive staining for NCC (green, arrow). Scale bar, 10 µm. Data is representative of three independent experiments.

To study the developmental aspect of kidney organoids, we investigated the time‐course of kidney organoid development and related gene expression. By day 3, organoids grew to ≈80 µm in diameter, and by day 14, ≈400 µm in diameter (**Figure** **2**A). We further demonstrated that from day 0 to 14, select kidney developmental genes were upregulated (Figure S2A, Supporting Information): anterior intermediate mesoderm markers *LHX1* and *GATA3*, posterior intermediate mesoderm markers *HOXD11* and *EYA1*, and presomitic mesoderm (PSM) marker *TBX6*. Consistent with previous reports was the downregulation of PSM marker *T* during organoid development.^[^ ^18^, ^19^ ^]^ We also observed gradual increases in podocyte markers WT‐1 and nephrin. No significant changes were found in Wnt4 (Figure S2A, Supporting Information), which interestingly, was highly expressed in these Sca1^+^ Oct4^+^ cells prior to organoid formation. Strong endogenous Wnt expression likely explains why these Sca1^+^ Oct4^+^ cell‐derived organoids formed in the absence of CHIR99021, a GSK‐3 inhibitor used to induce canonical Wnt signaling during kidney organoid formation from human pluripotent stem cells (hPSCs).^[^ ^18^, ^19^, ^20^ ^]^

We subsequently investigated whether we could detect these renal lineages in the Sca1^+^ Oct4^+^ cell‐derived organoids. We induced differentiation in Sca1^+^ Oct4^+^ cell‐derived organoids by culturing the organoids in Medium C for ≤ 14 days, and then in APEL medium for 7–14 days. Using this method, we generated self‐organized kidney‐shaped organoids from single cells (Figure 2B). Within these differentiated Sca1^+^ Oct4^+^ cell‐derived organoids, we observed characteristics of kidney structures including cortex, medulla, renal column, and renal capsule (Figure 2C). Cryosection staining further showed a glomerulus‐like structure with nephrin positive cells and tubule‐like structure positively stained for distal tubular marker NCC (Figure 2D). We also observed podocytes (nephrin^+^) and endothelial cells (CD31^+^) in close apposition to each other in the cryosection staining (Figure S2B, Supporting Information, top). Notably, we observed a structure with two CD31^+^ arms seemingly extended toward nephrin^+^ cells (Figure S2B, Supporting Information, bottom). Because the isolated Sca1^+^ Oct4^+^ cells were able to develop self‐organized 3D organoids with upregulated expression of kidney developmental genes, we believe that they are kidney stem cells.

Taken together, our findings suggest that these differentiated Sca1^+^ Oct4^+^ cell‐derived organoids contain endothelial (CD31^+^), podocyte (nephrin^+^), and distal tubular (NCC^+^) lineages.

### 2.3 Monolayer Cultures of Differentiated Sca1^+^ Oct4^+^ Cells Reveal Functional Mini Kidney‐Like Structure and an Entire De Novo Nephron

**Figure 3:**
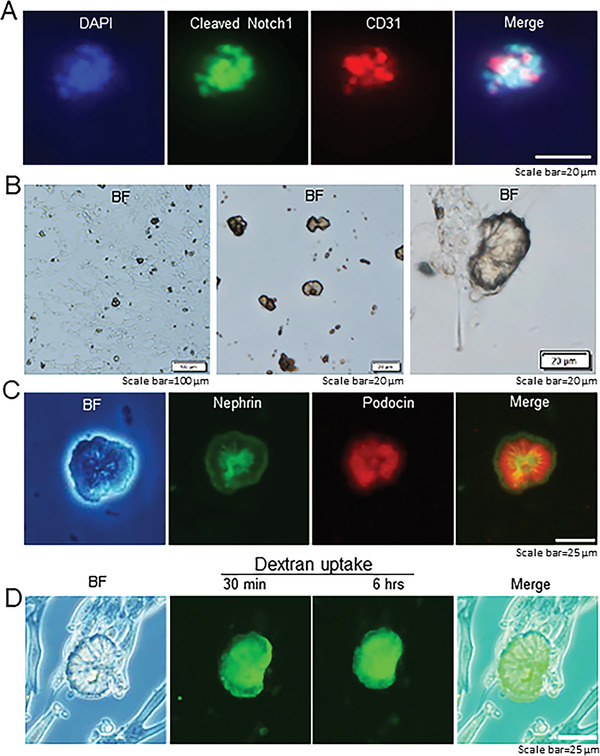
Generation of self‐organizing kidney‐shaped structures from the differentiated Sca1^+^ Oct4^+^ monolayer cultures. A) Comma‐shaped body with positive staining of cleaved‐Notch1 and CD31. Scale bar, 20 µm. B) Bright field (BF) of differentiation of the isolated Sca1^+^ Oct4^+^ cells and subsequent generation of self‐organized kidney‐shaped structures. Scale bar, 100, 20, and 20 µm. C) Expression of nephrin and podocin in self‐organizing kidney‐shaped structures assembled by differentiated Sca1^+^ Oct4^+^ cells. Scale bar, 25 µm. D) Uptake of 70 kDa FITC‐dextran by kidney‐shaped structures after 30 min and 6 h. Scale bar, 25 µm. Data is representative of three independent experiments.

To further characterize these Sca1^+^ Oct4^+^ cells, we induced differentiation by culturing monolayer Sca1^+^ Oct4^+^ cells in Medium D (DMEM/F12, 20% calf bovine serum, 1% penicillin streptomycin, 20 ng mL^−1^ stem cell factor, and 25 ng mL^−1^ bFGF) for ≤ 7 days, and subsequently in APEL medium for ≤ 30 days. Within these differentiated Sca1^+^ Oct4^+^ monolayer cultures, we detected ureteric bud (GATA3^+^) cells surrounded by a cap of metanephric mesenchyme (Six2^+^) cells (Figure S3A, Supporting Information); this suggests that progenitor cells for both collecting duct and nephrons, respectively, were present.^[^ ^21^, ^22^ ^]^ Comma‐ (cleaved‐Notch1^+^ CD31^+^) (**Figure** **3**A) and S‐shaped (cleaved‐Notch1^+^ LTL^+^) bodies were subsequently observed (Figure S3D, Supporting Information), indicating that clusters of Six2^+^ nephron progenitor cells had entered the early stages of Notch signaling‐mediated nephrogenesis.^[^ ^23^, ^24^, ^25^ ^]^

Notably, we observed the assembly of self‐organizing kidney‐shaped structures (Figure 3B). We examined the kidney‐shaped structures for evidence of renal cell types and found that they expressed podocyte markers nephrin and podocin (Figure 3C). Importantly, these kidney‐shaped structures selectively endocytosed 70 kDa FITC‐dextran after 30 min of exposure (Figure 3D); their ability to uptake dextran leads us to believe that proximal tubules were present.^[^ ^18^ ^]^ Our observations of these kidney‐shaped bodies suggest that the isolated Sca1^+^ Oct4^+^ cells exhibit characteristics of glomerular and proximal tubular lineages upon differentiation.

**Figure 4:**
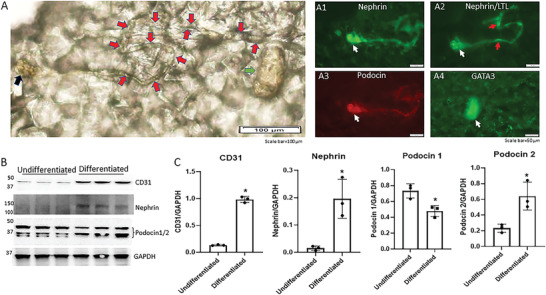
Generation of a self‐organizing de novo nephron from the differentiated Sca1^+^ Oct4^+^ monolayer cultures. A) Bright field image of self‐organizing organoid‐like structure from monolayer culture of kidney stem cells. The photo shows glomerulus‐like structure (black arrow), tubule‐like structure (red arrows), and collecting duct‐like structure (green arrow). Scale bar, 100 µm. A1) Glomerulus‐like structure stained with nephrin (white arrow). Scale bar, 50 µm. A2) Glomerulus‐like structure stained with nephrin (white arrow) and proximal tubule‐like structure stained with LTL (red arrows). Scale bar, 50 µm. A3) Glomerulus‐like structure stained with podocin (white arrow). Scale bar, 50 µm. A4) Collecting duct‐like structure stained with GATA3 (white arrow). Scale bar, 50 µm. ,C) Western blot analysis of CD31, nephrin, and podocin expression in undifferentiated and differentiated Sca1^+^ Oct4^+^ cells. GAPDH was used as a loading control. Data is representative of three independent experiments. Values represent the mean ± SD (*n* = 3). *p* < 0.05 versus control by 2‐tailed Student's *t* test.

Our findings encouraged us to further assess whether the isolated Sca1^+^ Oct4^+^ cells could form a self‐organizing nephron. Low and colleagues have demonstrated that hPSC‐derived kidney organoids give rise to patterned nephron segments;^[^ ^26^ ^]^ however, the de novo generation of an entire nephron has never been reported in mammals.^[^ ^27^ ^]^ To explore this, we cultured monolayer Sca1^+^ Oct4^+^ cells in Medium D for ≤ 7 days, and subsequently in APEL medium for ≤ 60 days. Remarkably, we observed the de novo assembly of a self‐organizing nephron inside of a ≈2 mm tissue structure (**Figure** **4**A). Within the self‐organizing nephron, we detected a glomerulus (nephrin^+^/podocin^+^, Figure 4A1,A3, white arrow), proximal tubule (LTL^+^, Figure 4A2, red arrow), and collecting duct (GATA3^+^, Figure 4A4, white arrow). We were unable to fluorescently label the loop of Henle and distal convoluted tubule with their respective markers, possibly due to the unfavorable permeability of the ≈2 mm tissue. However, cell lineages for both distal convoluted tubules (LTL^+^ ECAD^+^) and loops of Henle (UMOD^+^) were observed in differentiated Sca1^+^ Oct4^+^ monolayer cultures (Figure S3B,C, Supporting Information). An LTL^+^ proximal tubule‐like structure was also found in close proximity to an S‐shaped body (cleaved‐Notch1^+^ LTL^+^) (Figure S3D, Supporting Information). These findings suggest that self‐mediated nephrogenesis had occurred in these differentiated Sca1^+^ Oct4^+^ monolayer cultures. Our findings suggest that the isolated Sca1^+^ Oct4^+^ cells possess an inherent ability to orchestrate the de novo generation and assembly of a self‐organized nephron and its associated collecting duct.

Western blot analysis of CD31, nephrin, and podocin further confirmed the existence of glomerular cell types in these cultures (Figure 4B,C). The following protein expression patterns were observed. CD31, which was weakly detected in the isolated Sca1^+^ Oct4^+^ cells prior to differentiation, significantly increased during differentiation. Nephrin levels similarly increased during the differentiation of these Sca1^+^ Oct4^+^ cells, though no baseline expression of nephrin was detected in undifferentiated Sca1^+^ Oct4^+^ cells. Lastly, both differentiated and undifferentiated Sca1^+^ Oct4^+^ cells expressed podocin. Undifferentiated Sca1^+^ Oct4^+^ cells preferentially expressed podocin 1 (42 kDa), while differentiated Sca1^+^ Oct4^+^ cells preferentially expressed podocin 2 (≈35 kDa) (Figure 4B,C). This pattern is consistent with a previous study by Relle et al., which reported that podocin 2 is primarily detected in the glomerulus.^[^ ^28^ ^]^ Altogether, our findings suggest that these differentiated Sca1^+^ Oct4^+^ monolayer cultures contain glomerular and different tubular lineages. These findings further support our hypothesis that the isolated Sca1^+^ Oct4^+^ cells are kidney stem cells that have capacity of de novo nephrogenesis.

### 2.4 Sirt2 Modulates Canonical Wnt/*β*‐Catenin Signaling during the Kidney Organoid Development

**Figure 5:**
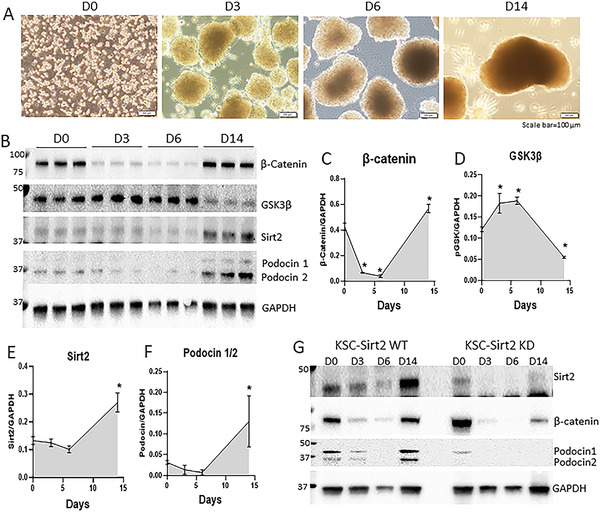
Sirt2 is required for dynamic change of canonical Wnt/*β*‐catenin signaling during kidney organoid development. A) Model of self‐organized kidney organoid development from single kidney stem cells. Scale bar, 100 µm. B) Western blot analysis of expression of *β*‐catenin, GSK3*β*, Sirt2, and podocin1/2 during kidney organoid development. GAPDH was used as loading control for quantitation. C–F) Quantification of gene expression for *β*‐catenin, GSK3*β*, Sirt2, and podocin1/2, respectively. G) Western blot analysis of Sirt2, *β*‐catenin, and podocin1/2 in KSC‐Sirt2 wt or KSC‐Sirt2 knockdown (KD) kidney organoids. *p < 0.05 versus day 0.

**Figure 6:**
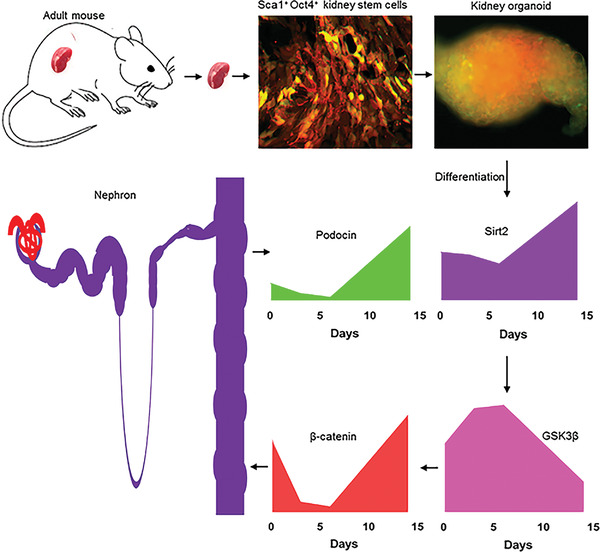
Schematic model of Sirt2‐mediated development of adult kidney stem cells‐derived kidney organoids. Adult kidney stem cells (KSCs) reside in the mouse kidney. KSCs were isolated using Sca1/Oct4 selection markers and labeled with mCherry and CD63‐GFP. KSCs formed self‐organized kidney organoid in culture mediated by Sirt2 expression. Sirt2 regulates GSK3*β* activity leading to a dynamic change of canonical Wnt/*β*‐catenin signaling and promotes the formation of KSCs‐derived kidney organoid and nephron development.

To explore the mechanism by which Sca1^+^ Oct4^+^ kidney stem cells develop self‐organized kidney organoids and de novo nephron structure in vitro, we studied the dynamic changes of canonical Wnt/*β*‐catenin signaling using our kidney organoid model as described above (**Figure** **5**A). The data showed that canonical Wnt signaling followed a biphasic pattern, with high activity in stem cells, decreased in early stage of organoid formation, and reactivated at day 14 of organoid development (Figure 5B,C). An opposite pattern was observed for the expression of GSK3*β* (Figure 5D), an inhibitor of canonical Wnt/*β*‐catenin signaling, during kidney organoid development. To study the role of Sirt2 in kidney organoid development, we investigated the expression of Sirt2 in our kidney organoid developmental model. We found that the expression of Sirt2 was low during the early stage of organoid development (d0‐d6), and dramatically increased at day 14 (Figure 5B,E). Interestingly, expression of podocin, a marker of podocytes located in the renal glomerulus, followed the similar pattern of Sirt2 expression during organoid development (Figure 5B,F). Knockdown of Sirt2 in kidney stem cells diminished the dynamic changes of canonical Wnt/*β*‐catenin signaling and impaired organoid development associated with decreased expression of podocin (Figure 5G; Figure S4, Supporting Information). Therefore, our findings strongly suggest that adult mouse kidney stem cells are capable of initiating Sirt2‐mediated dynamic changes of the canonical Wnt/*β*‐catenin signaling during kidney organoid development. A schematic developmental model of kidney stem cell‐derived kidney organoid labeled with mCherry/GFP is illustrated in **Figure** **6**.

We also measured Sit1 and Sirt3 protein expression during the kidney organoid formation. Unlike Sirt2, expression of Sirt1 and Sirt 3 was downregulated from day 0 to day 14 during the development of KSC‐derived kidney organoids (Figure S5, Supporting Information). This finding suggests that Srit1 and Srit3 may not play an essential role in the development of KSC‐derived kidney organoids.

## 3 Discussion

Kidney stem cells (KSCs) and KSCs‐derived kidney organoids could provide unprecedented opportunities for studying the mammalian kidney development and precision translational medicine. However, whether there exist adult kidney stem cells remains extensively debated due to a lack of successful attempts for using these cells to generate kidney organoids. Hence, we developed a comprehensive method and demonstrated that stem cells indeed reside in the adult mouse kidney, which is supported by the following evidence. 1) Kidney stem cells expressed a variety of common stem cell markers and renal markers (Figure 1C). 2) These kidney stem cells formed self‐organized 3D kidney organoids, which contain integrated glomerular structure with networks of capillary‐like structure and podocytes (Figure S2B, Supporting Information). 3) Differentiation of monolayer kidney stem cells developed self‐organized kidney‐shaped structures that co‐expressed nephrin and podocin, markers of glomerulus, and selectively endocytosed dextran (Figure 3C,D). 4) Comma‐ and S‐shaped bodies that expressed activated‐Notch1 were present during this formation (Figure 3A; Figure S3D, Supporting Information). 5) This is consistent with current knowledge that Notch signaling plays a key role in nephrogenesis during the mouse embryo development.^[^ ^25^, ^27^ ^]^ (6) Finally, we demonstrated a de novo generation of an entire nephron structure from differentiated monolayer cultures of kidney stem cells (Figure 4A).

Kidney organoids have been generated from human pluripotent stem cells (hPSCs).^[^ ^20^, ^26^ ^]^ However, the de novo generation of an entire nephron or a whole glomerulus using stem cells has never been reported.^[^ ^27^ ^]^ Canonical Wnt signaling is required for the initial stages of nephron formation in which expression of Wnt4 in the nephrogenic cap leads mesenchyme to epithelium transformation.^[^ ^29^ ^]^ Dynamic regulation of canonical Wnt signaling is also required for proper nephron development,^[^ ^30^ ^]^ and inhibition of canonical Wnt signaling blocks nephron progenitor cell proliferation.^[^ ^31^ ^]^ CHIR99021, an inhibitor of GSK‐3, is commonly used to manipulate canonical Wnt signaling for induction of metanephric mesenchyme in hPSCs‐derived kidney organoids.^[^ ^18^, ^20^ ^]^ In contrast, kidney stem cell‐derived organoids expressed different mesoderm markers during the development of self‐organizing kidney organoids (Figure S2A, Supporting Information), suggesting that the endogenous self‐regulated canonical Wnt signaling may play a role in the organoid formation. Indeed, we found that a dynamic regulation of canonical Wnt signaling occurred in a biphasic pattern during the formation of kidney stem cell‐derived organoids, with high activity in stem cells, diminished in early stage, and reactivated at day 14 of development (Figure 5A,B). Such dynamic changes of canonical the Wnt signaling are also found during the dendritic growth of adult‐born hippocampal neurons.^[^ ^32^ ^]^

A previous study has demonstrated that Sirt2 is upregulated during mouse embryonic stem cells (ESCs) differentiation and Sirt2 deficiency induces activation of GSK3*β*.^[^ ^33^ ^]^ In this study, we showed that the expression of Sirt2 was low at the early stage and was upregulated at day 14 of kidney organoid development associated with increase in podocin expression, a marker of podocytes of glomerulus (Figure 5B). Interestingly, the activity of GSK3*β* was also shown in biphasic pattern, which was opposite to the activity of *β*‐catenin. These findings strongly suggest that Sirt2 is differentially upregulated during kidney organoid development, which precisely modulates the dynamic changes of canonical Wnt signaling during organoid formation. High level of *β*‐catenin observed in the kidney stem cells is likely, at least in part, due to strong endogenous expression of Wnt4 (Figure 1C). Consistent with our findings, a recent study showed that Wnt4‐deficient organoids failed to undergo mesenchymal to epithelial transformation.^[^ ^34^ ^]^ Additionally, Kim and colleagues have shown that Sirt2 is required for efficient reprogramming of mouse embryonic fibroblasts toward pluripotency.^[^ ^35^ ^]^ They demonstrated that depletion of Sirt2 impaired reprogramming efficiency through activation of senescence marker p16. Hence, our findings strongly suggest that the crosstalk between Sirt2‐modulated dynamic changes of the canonical Wnt signaling and Sirt2‐mediated cell reprograming towards pluripotency may play a critical role in determining the proper lineage commitment of kidney stem cells during kidney development. This assumption is supported by our results that knockdown of Sirt2 in kidney stem cells blocked biphasic pattern of canonical Wnt/*β*‐catenin signaling and impaired the development of organoids leading to a decrease in podocin expression. This finding fundamentally advances our outstanding of mammalian kidney development and ex vivo kidney organoid generation. The limitations of current study were that we primarily focused on proof of concept and that the model of KSCs‐derived kidney organoids was mainly able to develop glomerulus‐like structure with few nephrons’ tubular segments. The nephron tubular segments were observed in the monolayer cultures of KSCs. A 3‐D culture of KSCs may help improve the development of glomerular structure with renal tubular segments. Additionally, implication of KSCs in the kidney disease models is highly desired in our future study.

## 4 Conclusion

In summary, we believe we have isolated stem cells from the adult mouse kidney that possess the capacity of de novo formation of kidney organoids. Culturing these cells led to generation of several kidney‐like structures containing nephron segments and lineages: i) self‐organized 3D kidney organoids with complex multicellular constructs, ii) self‐organized nephron with integrated segments and an associated collecting duct, and iii) functionally mature renal tubules and self‐organized kidney‐shaped bodies that endocytosed dextran. While our characterizations of these cells are unable to distinguish between stem cells and progenitor cells, this does not weaken our argument that stem cells exist in the adult mouse kidney. Rather, the presence of ureteric bud (GATA3^+^) and metanephric mesenchyme (Six2^+^) progenitor cells in our differentiated monolayer cultures supports the presence of stem cells in the adult mouse kidney.^[^ ^21^, ^22^, ^27^ ^]^ Thus, this study provides a convincing proof of concept that KSCs can form self‐organizing 3D organoids. Altogether, these findings have the potential to open new avenues for developing novel strategies for kidney regeneration.

## 5 Experimental Section

The objective of the study was to isolate kidney stem cells from adult mouse kidney. It was hypothesized that adult kidney stem cells were Sca1^+^ Oct4^+^ cells resided in the adult kidney. The kidney stem cells should have the capacity to generate kidney organoids under typical culture conditions in vitro. Sample sizes were not predetermined using power analysis. All independent experiments were conducted using biological replicates as described in the text. All experiments were performed in a standard laboratory setting.

### Isolation of Kidney Stem Cells (KSCs) from Mouse Kidney

All animal procedures were conducted according to guidelines provided by the National Institutes of Health and the Institute of Laboratory Animal Resources, National Research Council. The University of Tennessee Health Science Center's Animal Care and Use Committee approved all animal studies (Protocol number: 18‐030). Wild‐type C57BL/6 mice (3‐month‐olds with no prior kidney injury) were used for this study. Whole mouse kidneys were dissected and used for the isolation of kidney stem/progenitor cells, as described previously.^[^ ^36^ ^]^

### Cell Culture and siRNA Transfection

Single cells were filtered through a 10 µm cell strainer (PluriSelect, SKU43‐50010‐50) and cultured in serum‐free Medium A (DMEM/F12, 0% FBS and 1% penicillin streptomycin) for 24 h to deplete non‐multipotent cells. Remaining cells were cultured in Medium B (DMEM/F12, 10% FBS and 1% penicillin streptomycin) for up to 7 days for further selection. Surviving cells were maintained in Medium C (DMEM/F12, 20% FBS, 1% penicillin streptomycin, 20 ng mL^−1^ stem cell factor (R&D, 255‐SC‐010), and 25 ng mL^−1^ bFGF (R&D, 233‐FB‐025) and subsequently tested for mycoplasma infection (R&D, CUL001B). Sca1^+^ cells were isolated from these cultures using a mouse Sca1^+^ selection kit (STEMCELL Technologies, 18 756). These Sca1^+^ cells were further purified by flow cytometry, and ≈97% of sorted Sca1^+^ cells were confirmed Sca1^+^ Oct4^+^ with PerCP‐Sca1 (Fisher Scientific, #50‐158‐66) and AF‐488‐OCT4 (Fisher Scientific, #NB1002379) antibodies. Cell sorting was performed on a BD Biosciences FACSAria, and assessment of Sca1^+^ Oct4^+^ expression was performed on a Propel/Bio‐Rad Yeti/ZE5 at the University of Tennessee Health Science Center's flow core facility. Adipocyte and osteogenic differentiation studies were performed using commercial kits (STEMCELL Technologies, #0 5507 and #0 5504) to assess the multipotency of these Sca1^+^ Oct4^+^ cells. For the generation of kidney organoids, cells (≈1×10^6^) were plated in a 10 cm regular petri dish and cultured for up to 14 days in Medium C. Kidney organoids were subsequently differentiated in APEL medium (STEMCELL Technologies, #0 5270) containing antibiotic‐antimycotic (1 ml per 50 ml of APEL; Gibco, #15 240 096) for 7–14 days. For the differentiation of monolayer cells, cells were cultured in medium D (DMEM/F12, 20% calf bovine serum, 1% penicillin streptomycin, 20 ng mL^−1^ stem cell factor, and 25 ng mL^−1^ bFGF) for up to 7 days, and then cultured in the APEL medium for up to 60 days. To knockdown Sirt2, mouse Sirt2 siRNA (Thermo Fisher Scientific, #AM16708) and negative control (Sigma, #SIC001‐10NMOL) were transfected into kidney stem cells (passage 66) with Lipofectamine 3000 transfection reagent (Thermo Fisher Scientific, #L3000008) at the final concentration of 5nM following the manufacture's instruction for 18 h. Then, transfected cells were cultured in Medium C for 48 h and used for kidney organoids study. The efficiency of Sirt2 knockdown was evaluated by Western blot analysis.

### Cryosectioning

Select organoids were sectioned prior to immunofluorescence staining. Such samples were fixed in 4% paraformaldehyde for 30 min at 4 °C and washed in PBS 3 times. Fixed organoids were subsequently cryopreserved in 30% sucrose overnight at 4 °C and embedded in OCT the following day. Samples were then frozen on dry ice and stored at −80 °C prior to cutting 10 µm cryosections with a microtome cryostat (MICROM International, Germany).

### Immunohistochemistry

The immunohistochemical procedure was provided in the recent reports.^[^ ^37^, ^38^, ^39^, ^40^, ^41^ ^]^ Briefly, monolayer cells and kidney organoids in cell culture flasks were fixed in 4% paraformaldehyde for 20 min at 4 °C, followed by 3 PBS washes. Samples were blocked with 5% goat serum in 0.1% Triton X‐100/PBS for 2–3 h at room temperature and subsequently incubated with primary antibodies overnight at 4 °C. The following antibodies and dilutions were used: goat anti‐nephrin (1:200, R&D AF3159), rabbit anti‐NCC (1:100, StressMarq SPC‐402), rabbit anti‐CD31 (1:200, Abcam ab28364), rabbit anti‐podocin (1:200, Abcam ab50339), rabbit anti‐Six2 (1:200, Proteintech #11562‐1‐AP), goat anti‐GATA‐3 (1:200, R&D AF2605‐SP), mouse anti‐E‐cadherin (1:200, Invitrogen #13‐1900), rabbit anti‐cleaved‐Notch1 (1:100, Cell Signaling #4147S), rabbit anti‐UMOD (1:100, Abcam ab207170), and LTL‐fluorescein (1:1000, Vector Laboratories FL‐1321). After 3 10‐min washes with 0.1% Triton X‐100/PBS, samples were incubated with secondary antibodies for 1 h at room temperature. Corresponding Alexa Fluor 448, 555, or 647 secondary antibodies (Invitrogen) were used at 1:1000. Images were captured using an Olympus IX73 fluorescence microscope (Japan).

### qRT‐PCR Analysis

The qRT‐PCR procedure was described in the recent studies.^[^ ^42^, ^43^, ^44^ ^]^ Briefly, total RNA was isolated from cells using a RNeasy Mini Kit (Qiagen, Germany, 74 104). For quantitative RT‐PCR (qRT‐PCR), 1.0 µg total RNA was reverse transcribed using an iScript cDNA synthesis kit (Bio‐Rad, 1 708 891). Each 20 µL qRT‐PCR reaction contained 2 µL of cDNA, 300 × 10^−9^ m each primer, and 1× iQ SYBR Green (Bio‐Rad, 1 708 880). qRT‐PCR analyses were performed on a CFX96 Real‐Time PCR Detection Systems (Bio‐Rad) machine. Relative expression values were evaluated with the 2^–ΔΔ^Ct method using GAPDH as the housekeeping gene. The sequences of primers used for qRT‐PCR are listed in Table S1 in the Supporting Information.

### Western Blot Analysis

The western blot procedure was provided in the recent studies.^[^ ^45^, ^46^, ^47^, ^48^, ^49^ ^]^ Briefly, samples from cells were lysated using M‐per mammalian extraction buffer (ThermoFisher, 78 501). For electrophoresis, 20 µg of cell lysates were loaded onto Expressplus Page 4–12% gel (GenScript, M42012). Proteins were separated at 120 V for 1 h and transferred to nitrocellulose membrane using Trans‐Blot Turbo (BioRad). Membranes were blocked with 5% BSA blocking buffer in TBST (Fisher Scientific, BP9706100) for 2 h and then incubated with primary antibody (CD31 1:500, Abcam ab28364; podocin 1:1000, Abcam ab50339; nephrin 1:1000, R&D AF3159‐SP; Sirt2 1:500, Cell Signaling #12 672, *β*‐catenin 1:1000, Cell Signaling #9562; GSK3*β* 1:1000, Cell Signaling #9315, and GAPDH 1:1000, Cell Signaling #2118) with gentle agitation overnight at 4 °C. After three washes with TBST (1×15 min and 2×5 min), membranes were incubated with secondary antibody in 5% BSA blocking buffer at room temperature for 1 h. Membranes were then washed 3 times (1×15 min and 2×5 min), subjected to ECL (BioRad), and analyzed with a BioRad ChemiDoc MP imaging system.

### Functional Analysis of Proximal Tubules

For dextran uptake assay, 1 µg mL^−1^ of 70000 MW FITC‐dextran (Sigma, 54 702) was cultured with cells for up to 24 h. Images were taken with an Olympus IX73 fluorescence microscope (Japan).

The key resources are summarized in Table S2 in the Supporting Information.

### Statistical Analysis

Differences between two groups were evaluated by 2‐tailed Student's *t* test. All values are expressed as means ± SD. *N* = 3 independent experiments for each statistical snalysis. Signifcance was set as nonsignivicant *p*>0.05 and significant *p < 0.05 versus control. All computations were performed using GraphPad Prism 8 (GraphPad Software Inc. La Jolla, CA, USA). No methods were used to predetermine the sample size. The experiments were not randomized, and the investigators were not blinded to allocation during experiments and outcome assessment.

## Conflict of Interest

The authors declare no conflict of interest.

## Supporting information

Supporting information

## Data Availability

The data that support the findings of this study are available from the corresponding author upon reasonable request.

## References

[1] P. T. Lee , H. H. Lin , S. T. Jiang , P. J. Lu , K. J. Chou , H. C. Fang , Y. Y. Chiou , M. J. Tang , Stem Cells 2010, 28, 573. 20099318 10.1002/stem.310

[2] B. Dekel , L. Zangi , E. Shezen , S. Reich‐Zeliger , S. Eventov‐Friedman , H. Katchman , J. Jacob‐Hirsch , N. Amariglio , G. Rechavi , R. Margalit , Y. Reisner , J. Am. Soc. Nephrol. 2006, 17, 3300. 17093069 10.1681/ASN.2005020195

[3] N. Barker , M. B. Rookmaaker , P. Kujala , A. Ng , M. Leushacke , H. Snippert , M. van de Wetering , S. Tan , J. H. Van Es , M. Huch , R. Poulsom , M. C. Verhaar , P. J. Peters , H. Clevers , Cell Rep. 2012, 2, 540. 22999937 10.1016/j.celrep.2012.08.018

[4] H. C. Park , K. Yasuda , M. C. Kuo , J. Ni , B. Ratliff , P. Chander , M. S. Goligorsky , Am. J. Physiol. Renal. Physiol. 2010, 298, F1254. 20200095 10.1152/ajprenal.00406.2009 PMC2867407

[5] J. A. Oliver , O. Maarouf , F. H. Cheema , T. P. Martens , Q. Al‐Awqati , J. Clin. Invest. 2004, 114, 795. 15372103 10.1172/JCI20921 PMC516259

[6] E. Ronconi , C. Sagrinati , M. L. Angelotti , E. Lazzeri , B. Mazzinghi , L. Ballerini , E. Parente , F. Becherucci , M. Gacci , M. Carini , E. Maggi , M. Serio , G. B. Vannelli , L. Lasagni , S. Romagnani , P. Romagnani , J. Am. Soc. Nephrol. 2009, 20, 322. 19092120 10.1681/ASN.2008070709 PMC2637058

[7] H. Iwatani , T. Ito , E. Imai , Y. Matsuzaki , A. Suzuki , M. Yamato , M. Okabe , M. Hori , Kidney Int. 2004, 65, 1604. 15086898 10.1111/j.1523-1755.2004.00561.x

[8] K. Hishikawa , T. Marumo , S. Miura , A. Nakanishi , Y. Matsuzaki , K. Shibata , T. Ichiyanagi , H. Kohike , T. Komori , I. Takahashi , O. Takase , N. Imai , M. Yoshikawa , T. Inowa , M. Hayashi , T. Nakaki , H. Nakauchi , H. Okano , T. Fujita , J. Cell Biol. 2005, 169, 921. 15967813 10.1083/jcb.200412167 PMC2171631

[9] G. A. Challen , I. Bertoncello , J. A. Deane , S. D. Ricardo , M. H. Little , J. Am. Soc. Nephrol. 2006, 17, 1896. 16707564 10.1681/ASN.2005111228

[10] B. Bussolati , S. Bruno , C. Grange , S. Buttiglieri , M. C. Deregibus , D. Cantino , G. Camussi , Am J Pathol 2005, 166, 545. 15681837 10.1016/S0002-9440(10)62276-6 PMC1602314

[11] C. Sagrinati , G. S. Netti , B. Mazzinghi , E. Lazzeri , F. Liotta , F. Frosali , E. Ronconi , C. Meini , M. Gacci , R. Squecco , M. Carini , L. Gesualdo , F. Francini , E. Maggi , F. Annunziato , L. Lasagni , M. Serio , S. Romagnani , P. Romagnani , J. Am. Soc. Nephrol. 2006, 17, 2443. 16885410 10.1681/ASN.2006010089

[12] S. Gupta , C. Verfaillie , D. Chmielewski , S. Kren , K. Eidman , J. Connaire , Y. Heremans , T. Lund , M. Blackstad , Y. Jiang , A. Luttun , M. E. Rosenberg , J. Am. Soc. Nephrol. 2006, 17, 3028. 16988061 10.1681/ASN.2006030275

[13] R. Morizane , J. V. Bonventre , Trends Mol. Med. 2017, 23, 246. 28188103 10.1016/j.molmed.2017.01.001 PMC5442988

[14] C. Vigneau , K. Polgar , G. Striker , J. Elliott , D. Hyink , O. Weber , H. J. Fehling , G. Keller , C. Burrow , P. Wilson , J. Am. Soc. Nephrol. 2007, 18, 1709. 17475814 10.1681/ASN.2006101078

[15] Y. Wang , J. Yang , T. Hong , X. Chen , L. Cui , Ageing Res. Rev. 2019, 55, 100961. 31505260 10.1016/j.arr.2019.100961

[16] A. Bahrami , F. Amerizadeh , S. ShahidSales , M. Khazaei , M. Ghayour‐Mobarhan , H. R. Sadeghnia , M. Maftouh , S. M. Hassanian , A. Avan , J. Cell. Biochem. 2017, 118, 1979. 28109136 10.1002/jcb.25903

[17] K. Berger , M. J. Moeller , Semin. Nephrol. 2014, 34, 394. 25217268 10.1016/j.semnephrol.2014.06.006

[18] M. Takasato , P. X. Er , H. S. Chiu , B. Maier , G. J. Baillie , C. Ferguson , R. G. Parton , E. J. Wolvetang , M. S. Roost , S. M. C. d. S. Lopes , M. H. Little , Nature 2016, 536, 238. 10.1038/nature17982 27120161

[19] A. Taguchi , Y. Kaku , T. Ohmori , S. Sharmin , M. Ogawa , H. Sasaki , R. Nishinakamura , Cell Stem Cell 2014, 14, 53. 24332837 10.1016/j.stem.2013.11.010

[20] M. Takasato , P. X. Er , H. S. Chiu , M. H. Little , Nat. Protoc. 2016, 11, 1681. 27560173 10.1038/nprot.2016.098 PMC5113819

[21] M. H. Little , Cell Stem Cell 2008, 2, 191. 18371440 10.1016/j.stem.2008.02.007

[22] M. C. Labastie , M. Catala , J. M. Gregoire , B. Peault , Kidney Int. 1995, 47, 1597. 7643528 10.1038/ki.1995.223

[23] M. H. Little , Cell Death Discovery 2016, 2, 16053. 27551541 10.1038/cddiscovery.2016.53 PMC4979446

[24] B. McCright , Curr. Opin. Nephrol. Hypertens. 2003, 12, 5. 12496659 10.1097/00041552-200301000-00002

[25] M. Mukherjee , E. Fogarty , M. Janga , K. Surendran , Biomolecules 2019, 9, 692. 10.3390/biom9110692 PMC6920979 31690016

[26] J. H. Low , P. Li , E. G. Y. Chew , B. Zhou , K. Suzuki , T. Zhang , M. M. Lian , M. Liu , E. Aizawa , C. Rodriguez Esteban , K. S. M. Yong , Q. Chen , J. M. Campistol , M. Fang , C. C. Khor , J. N. Foo , J. C. Izpisua Belmonte , Y. Xia , Cell Stem Cell 2019, 25, 373. 31303547 10.1016/j.stem.2019.06.009 PMC6731150

[27] S. E. Quaggin , J. A. Kreidberg , Development 2008, 135, 609. 18184729 10.1242/dev.001081

[28] M. Relle , H. Cash , C. Brochhausen , D. Strand , J. Menke , P. R. Galle , A. Schwarting , Mod. Pathol. 2011, 24, 1101. 21499232 10.1038/modpathol.2011.58 PMC3182839

[29] K. Stark , S. Vainio , G. Vassileva , A. P. McMahon , Nature 1994, 372, 679. 7990960 10.1038/372679a0

[30] J. S. Park , M. T. Valerius , A. P. McMahon , Development 2007, 134, 2533. 17537789 10.1242/dev.006155

[31] C. N. Kamei , T. F. Gallegos , Y. Liu , N. Hukriede , I. A. Drummond , Development 2019, 146, dev168294. 31036548 10.1242/dev.168294 PMC6503981

[32] J. Heppt , M. T. Wittmann , I. Schaffner , C. Billmann , J. Zhang , D. Vogt‐Weisenhorn , N. Prakash , W. Wurst , M. M. Taketo , D. C. Lie , EMBO J. 2020, 39, 104472. 10.15252/embj.2020104472 PMC7604596 32929771

[33] X. Si , W. Chen , X. Guo , L. Chen , G. Wang , Y. Xu , J. Kang , PLoS One 2013, 8, 76699. 10.1371/journal.pone.0076699 PMC3800056 24204656

[34] Z. Tan , J. Shan , A. Rak‐Raszewska , S. J. Vainio , Sci. Rep. 2018, 8, 16618. 30413738 10.1038/s41598-018-34995-3 PMC6226521

[35] A. Y. Kim , E. M. Lee , E. J. Lee , J. H. Kim , K. Suk , E. Lee , K. Hur , Y. J. Hong , J. T. Do , S. Park , K. S. Jeong , Cell Death Dis. 2018, 9, 893. 30166528 10.1038/s41419-018-0920-3 PMC6117269

[36] X. Han , Z. Sun , Hypertension 2020, 75, 1233. 32223380 10.1161/HYPERTENSIONAHA.120.14642 PMC7805580

[37] R. Feng , M. Ullah , K. Chen , Q. Ali , Y. Lin , Z. Sun , J. Extracell. Vesicles 2020, 9, 1783869. 32939234 10.1080/20013078.2020.1783869 PMC7480600

[38] D. Gao , S. Wang , Y. Lin , Z. Sun , Redox Biol. 2020, 37, 101692. 32863229 10.1016/j.redox.2020.101692 PMC7476318

[39] Y. Lin , Z. Sun , J. Cell. Physiol. 2022, 237, 720. 34368951 10.1002/jcp.30541 PMC8810603

[40] Y. Lin , Z. Sun , Diabetes 2015, 64, 4298. 26340932 10.2337/db15-0066 PMC4657580

[41] Y. Lin , Z. Sun , Diabetes 2015, 64, 1444. 25377875 10.2337/db14-0632 PMC4375073

[42] M. Ullah , Z. Sun , J. Gerontol., Ser. A 2019, 74, 1396. 10.1093/gerona/gly261 PMC6696722 30452555

[43] D. Jung , Y. Xu , Z. Sun , Oncotarget 2017, 8, 46745. 28657902 10.18632/oncotarget.18608 PMC5564520

[44] K. Chen , Z. Sun , J. Mol. Med. 2019, 97, 1615. 31630227 10.1007/s00109-019-01841-6 PMC7055468

[45] K. Chen , Z. Sun , Redox Biol. 2021, 47, 102173. 34678656 10.1016/j.redox.2021.102173 PMC8577443

[46] K. Chen , S. Wang , Q. W. Sun , B. Zhang , M. Ullah , Z. Sun , Circ. Res. 2021, 128, 492. 33334122 10.1161/CIRCRESAHA.120.317348 PMC8782577

[47] Q. Wang , S. Wang , Z. Sun , Hypertension 2021, 78, 308. 34176284 10.1161/HYPERTENSIONAHA.121.17299 PMC8285030

[48] J. Y. Chen , R. An , Z. J. Liu , J. J. Wang , S. Z. Chen , M. M. Hong , J. H. Liu , M. Y. Xiao , Y. F. Chen , Acta Pharmacol. Sin. 2014, 35, 1121. 25088001 10.1038/aps.2014.61 PMC4155533

[49] D. Gao , Z. Zuo , J. Tian , Q. Ali , Y. Lin , H. Lei , Z. Sun , Hypertension 2016, 68, 1191. 27620389 10.1161/HYPERTENSIONAHA.116.07709 PMC5063709

