# Supplementary material for: Adult Mouse Kidney Stem Cells Orchestrate the De Novo Assembly of a Nephron via Sirt2‐Modulated Canonical Wnt/β‐Catenin Signaling
Source: Adv Sci (Weinh). 2022 Mar 22;9(15):2104034. doi: 10.1002/advs.202104034 (PMC9130916; doi:10.1002/advs.202104034)
Supplement: Supplementary file 1 — Supporting information [file ADVS-9-2104034-s001.pdf]

ADVANCED SCIENCE
Open Access

Supporting Information

for *Adv. Sci.*, DOI 10.1002/advs.202104034

Adult Mouse Kidney Stem Cells Orchestrate the De Novo Assembly of a Nephron via Sirt2-Modulated Canonical Wnt/β-Catenin Signaling

*Xiaobin Han and Zhongjie Sun**

Supporting Information

Adult mouse kidney stem cells orchestrate the *de novo* assembly of a nephron *via* Sirt2-modulated canonical Wnt/β-catenin signaling

*Xiaobin Han and Zhongjie Sun**

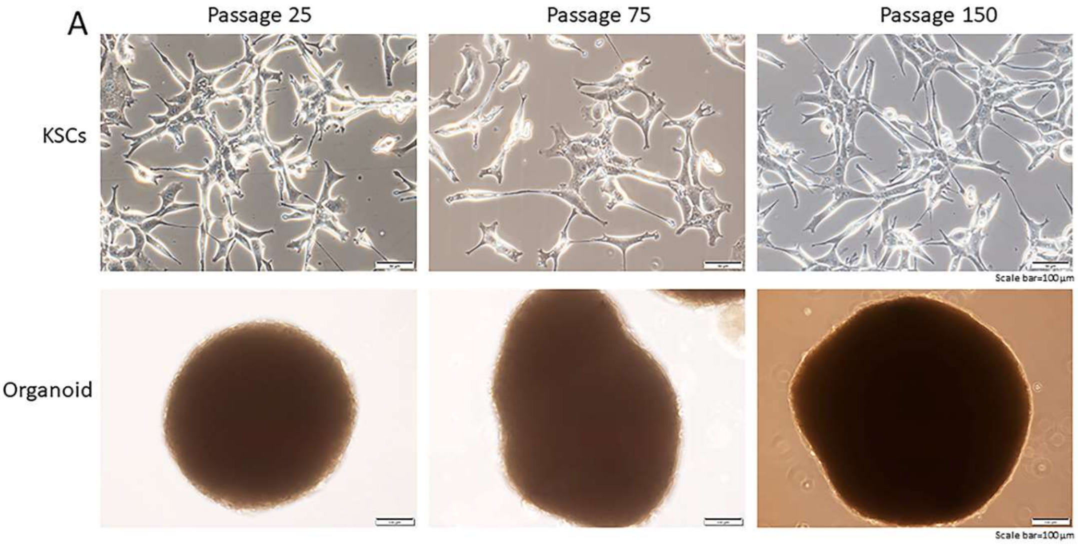

Figure S1. The isolated Sca1⁺ Oct4⁺ cells form self-organizing organoids. (**A**) Passages of Sca1⁺ Oct4⁺ cells and kidney organoid formations by each passage of Sca1⁺ Oct4⁺ cells, respectively. Scale bar, 100 μm.

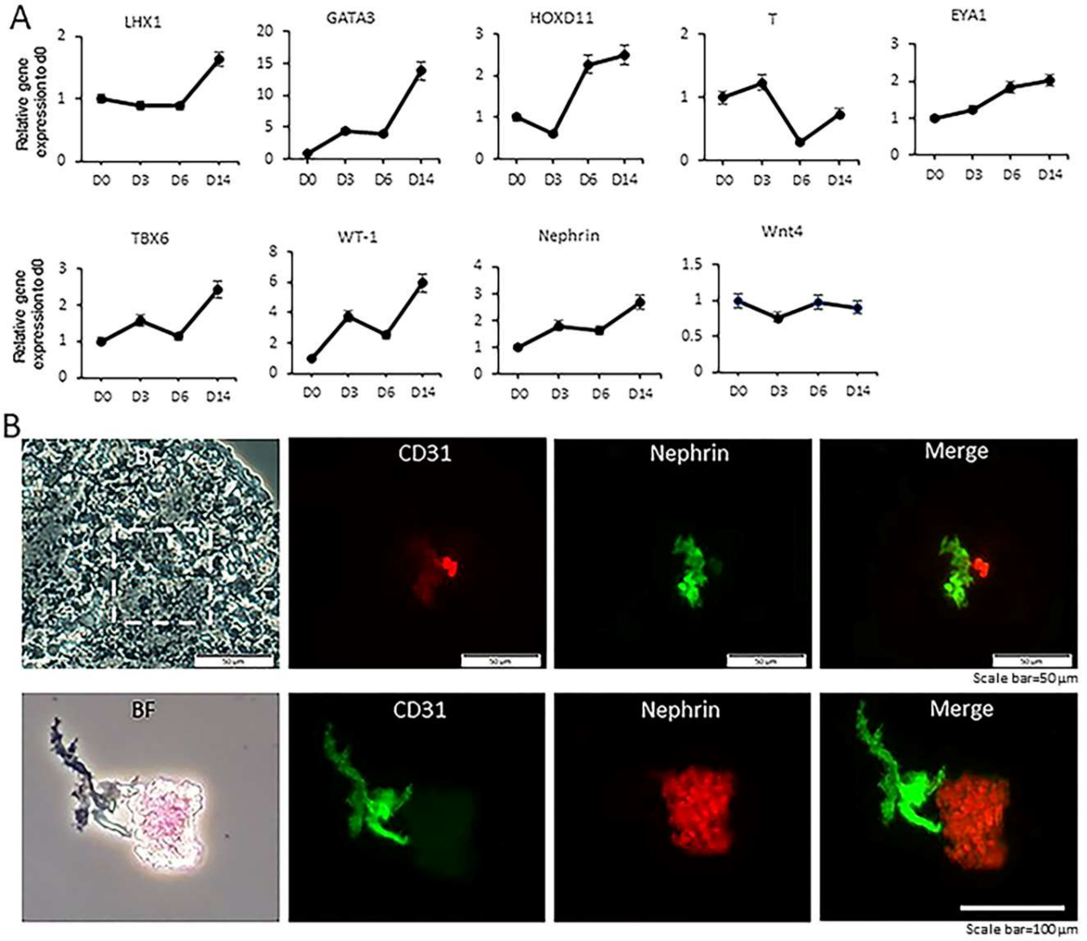

Figure S2. Kidney organoids derived from the isolated Sca1⁺ Oct4⁺ cells contain glomerular and vascular lineages. (A) Expression of kidney developmental genes in Sca1⁺ Oct4⁺ cell-derived kidney organoids at days 0, 3, 6, and 14. (**B**) Endothelial cells (CD31⁺) and podocytes (nephrin⁺) in close apposition to each other within a Sca1⁺ Oct4⁺ cell-derived kidney organoid (cryosections) (top). Scale bar, 50 µm. Glomerulus-like structure with two CD31⁺ arms seemingly extended toward nephrin⁺ cells (bottom). Scale bar, 100 µm. Data is representative of 3 independent experiments.

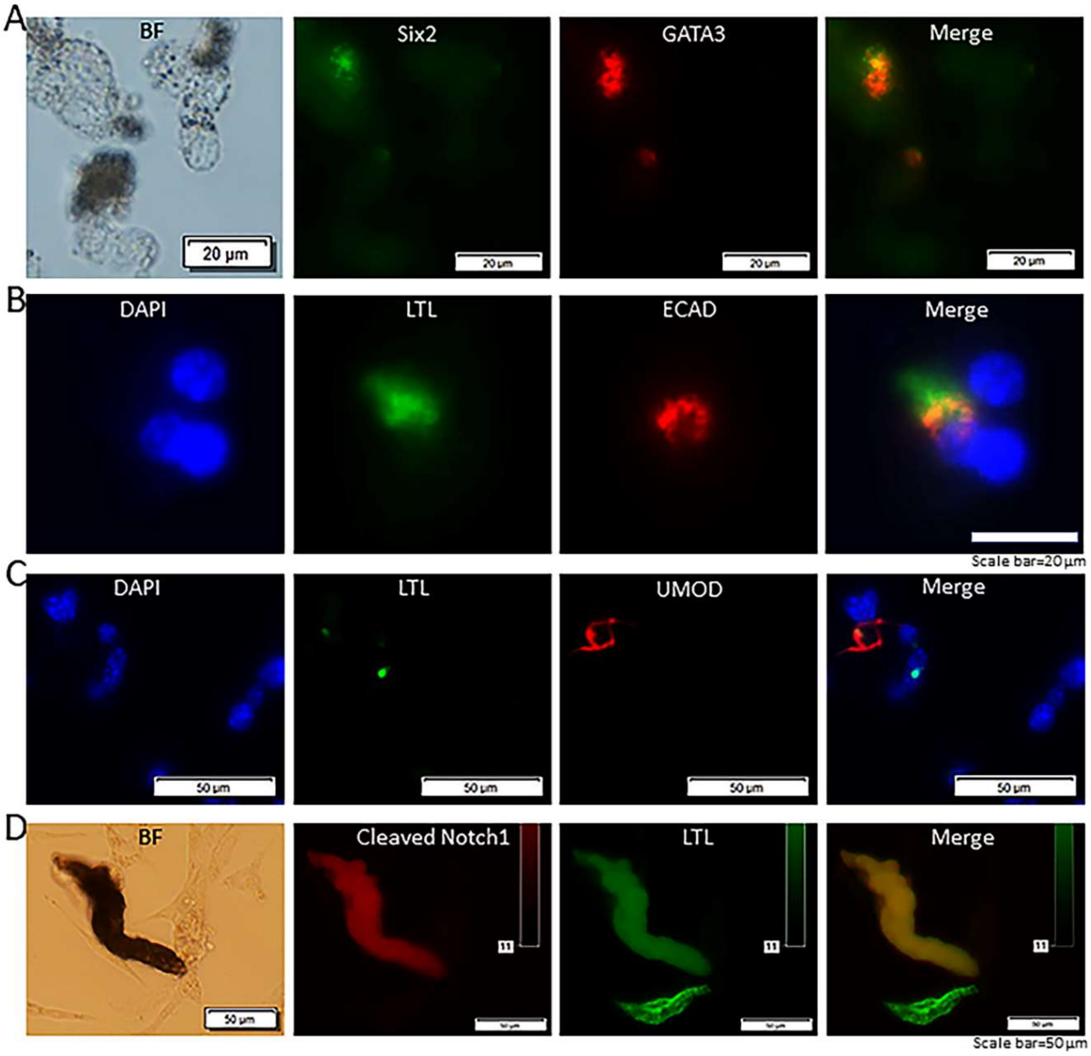

Figure S3. Detection of ureteric bud, metanephric mesenchyme, proximal tubule, and loop of Henle lineages in the differentiated Sca1$^+$ Oct4$^+$ monolayer cultures. A. GATA3$^+$ collecting duct progenitors surrounded by a cap of Six2$^+$ nephron progenitors. Scale bar, 20 µm. **B.** Co-localization of mature proximal tubular markers LTL and ECAD. Scale bar, 20 µm. **C.** Loop of Henle (UMOD$^+$) cell lineage observed in the differentiated Sca1$^+$ Oct4$^+$ monolayer cultures. Scale bar, 50 µm. **D.** S-shaped body with positive staining for cleaved-Notch1 and LTL alongside an LTL$^+$ proximal tubule-like structure. Scale bar, 50 µm.

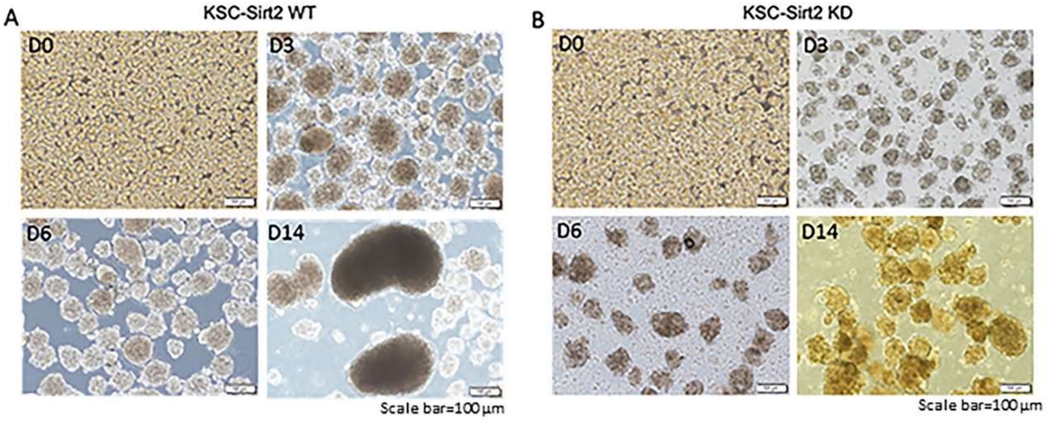

Figure S4. Sirt2 is required for dynamic change of canonical Wnt/β-catenin signaling during kidney organoid development. (**A**) Kidney organoids derived from KSC-Sirt2 wild type and KSC-Sirt2 knockdown cells. Scale bar, 100 µm.

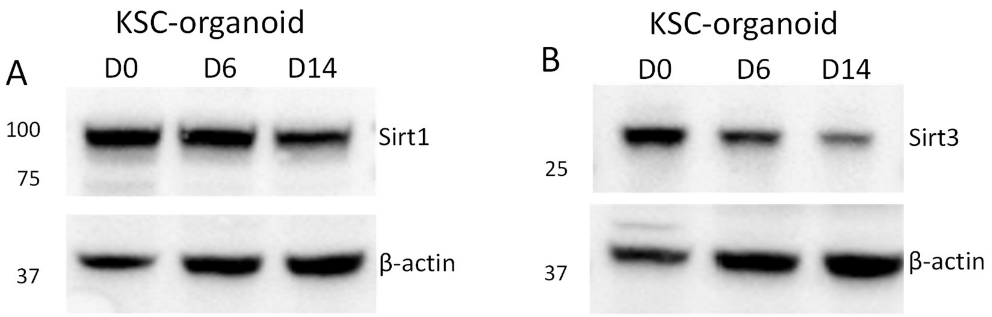

Figure S5. **Expression of Sirt1 and Sirt3 is downregulated during the development of KSC-organoids.** (**A**). Western blot analysis of Sirt1 protein expression at day 0, 6, and 14 of the development of KSCs-derived kidney organoids. (**B**). Western blot analysis of Sirt3 protein expression at day 0, 6, and 14 of the development of KSCs-derived kidney organoids. β-actin was used as loading control.

Table S1. Primers for qRT-PCR and RT-PCR.

Supplemental Table 1.		
Real-time PCR (qRT-PCR)		
Gene names	Forward sequence (5'-3')	Reverse sequence (5' 3')
T	AGGTACCCAGCTCTAAGGAA	AGTAGGTGGGCTGGCGTTAT
LHX1	ATCCTGGACCGTTTCCTCTTGA	TATCTTCCGCTTGCTTTGCCT
TBX6	AGCCTGAGCTTGGAGAACCA	TCAGCCTTGCCACTTGGCT
GATA3	TTAGGACTGGGCCTTTCGT	TCCAGGGCGAGGCGGTGT
HOXD11	TACGCGCTGCGCAGAAAGCT	TCGTCAAAGTCGTTCATGGCT
EYA1	TGCACAGCAGCCAAACTAT	ACTGTGCTTCACTGTCAGC
WT1	ACATCCGACTTCCAGACAGCACAC	TTGCAGCCAGACCTCTGAAATTCT
Wnt4	AGAACTGGAGAAGTG CTGTGACC	TGTATGTGGCTTGAACTGTGCATTCCG
Nephrin	TCCAGACACCTGACACAGCT	ACCCACTCACAGGTACTCA
GAPDH	CACCACCAACTGCTTAGCC	TGGCATGGACTGTGGTCA
RT-PCR		
Gene names	Forward sequence (5'-3')	Reverse sequence (5' 3')
Oct4	CACGAGTGGAAAGCAACTCA	AGATGGTGGTCTGGCTGAAC
Sca-1	CCATCAATTACCTGCCCCTA	CCATCAATTACCTGCCCCTA
CD133	ACCAACACCAAGAACAAGGC	GGAGCTGACTTGAATTGAGG
CD45	CCACCAGGGACTGACAAGTT	TGTAATTTGTTTGGGCACGA
CD34	ACCACAGACTTCCCCAACTG	CGGATTCCAGAGCATTTGAT
Cadherin-11	ATGGGGCACTGTTGTCCTGT	CCTCACCACCCCCTTCATCATCATAG
Pax-2	TCCCAGTGTCTCATCCATCA	GTTAGAGGCGCTGGAAACAG
WT-1	ACATCCGACTTCCAAGACAGCACAC	TTGCAGCCAGACCTCTGAAATTCTG
Wnt-4	AGAACTGGAGAAGTGTGGCTGTGACC	TGTATGTGGCTTGAACTGTGCATTCCG
GAPDH	ACGGCACAGTCAAGGCTGAG	GGAGGCCATGTAGACCATGAGG

Table S2. Key Resources

**REAGENT or RESOURCE**	SOURCE	IDENTIFIER
Antibodies		
NEPHRIN	R&D System	Cat# AF3159, RRID:AB_2155023
NCC	StressMarq	Cat# SPC-402
CD31	Abcam	Cat# ab28264, RRID:AB_726362
PODOCIN (NPSH2)	Abcam	Cat# ab50339, RRID:AB_882097
SIX2	Proteintech	Cat# 11562-1-AP, RRID:AB_2189084
GATA3	R&D System	Cat# AF2605, RRID:AB_2108571
E-Cadherin	Thermo Fisher Scientific	Cat# 13-1900, RRID:AB_2533005
Cleaved Notch1	Cell Signaling Technology	Cat# 4147, RRID:AB_2153348
UMOD	Abcam	Cat# 207170
Sirt2	Cell Signaling Technology	Cat# 12672, RRID:AB_2636961

β-catenin	Cell Signaling Technology	Cat# 9562, RRID:AB_331149
GSK3β	Cell Signaling Technology	Cat# 9315, RRID:AB_490890
GAPDH	Cell Signaling Technology	Cat# 2118, RRID:AB_561053
Sca1	Fisher Scientific	Cat#50-158-66
Anti-Oct4	Fisher Scientific	Cat#NB1002379
Goat anti-Rabbit IgG, Alexa Fluor 488	Thermo Fisher Scientific	Cat# A-11008, RRID:AB_143165
Goat anti-Rabbit IgG, Alexa Fluor 555	Thermo Fisher Scientific	Cat# A27039, RRID:AB_2536100
Donkey anti-Mouse IgG, Alexa Fluor 647	Thermo Fisher Scientific	Cat# A-31571, RRID:AB_162542
Donkey anti-Goat IgG, Alexa Fluor 488	Thermo Fisher Scientific	Cat# A32814, RRID:AB_2762838
Donkey anti-Goat IgG, Alexa Fluor 647	Thermo Fisher Scientific	Cat# A-21447, RRID:AB_2535864
Goat anti-Rabbit IgG, Secondary antibody, HRP	Thermo Fisher Scientific	Cat# 31460, RRID:AB_228341
Donkey anti-Goat IgG, Secondary antibody, HRP	Thermo Fisher Scientific	Cat# A15999, RRID:AB_2534673
**Biological Samples**		
Adult mouse kidney tissues	Zhongjie Sun Lab	
**Experimental Model: Cell line**		
Adult Mouse Kidney Stem Cells	Zhongjie Sun Lab	
**Chemicals, Peptides, and Recombinant Proteins**		
Recombinant Human SCF Protein	R&D System	Cat#255-SC-010
Recombinant Human FGF basic (146 aa) Protein	R&D System	Cat#233-FB-025
Phosphatase inhibitor cocktail 3 DMSO solution	Sigma	Cat#P0044
Protease inhibitor cocktail	Sigma	Cat#p8840
Fluorescein labeled Lotus Tetragonolobus Lectin (LTL)	Vector Lab	Cat#FL-1321
**Commercial Kits**		
MesenCult Osteogenic Stimulatory Kit (Mouse)	StemCell Technologies	Cat#05504
MesenCult Adipogenic Differentiation Kit (Mouse)	StemCell Technologies	Cat#05507
Intracellular Staining Flow cytometry Kit	Novus Biologicals	Cat#NBP2-29450
REDExtract-N-Amp PCR redayMix	Sigma	Cat#R4775
EasySep Mouse SCA1 Positive Selection Kit	StemCell Technologies	Cat#18756
Lipofectamine 3000 Transfection Reagent	Thermo Fisher Scientific	Cat#L3000008
iScript cDNA Synthesis Kit	Bio Rad	Cat#1708891
iQ SYBR Green Supermix	Bio Rad	Cat#1708882
SIRT2 siRNAs mouse	Thermo Fisher Scientific	Cat#AM16708

MISSION siRNA Universal Negative control #1	Sigma	Cat#SIC001-10NMOL
MycoProbe Mycoplasma Detection Kit	R&D System	Cat#CUL001B
**Software**		
GraphPad Prism 8	GraphPad	http://www.graphpad.com/; RRID:SCR_002798
**Other**		
Hibernate A Medium	BraniBits	Cat#SKU:HA
Ptotease Inhibitor cocktail	Cell Signaling Technology	Cat#5871
DTT (Dithiothreitol)	Cell Signaling Technology	Cat#7016
Collagenase, type II	Thermo Fisher Scientific	Cat#17101015
DMEM/F-12 medium	Thermo Fisher Scientific	Cat#1132003
ReleSR Enzyme-free human ES and iPS cell selection and passaging reagent	StemCell Technologies	Cat#05872
L-Glutamine	StemCell Technologies	Cat#07100
Alizarin Red S	Sigma	Cat#A5533-25G
Fetal Bovine Serum	VWR	Cat#97068-085
Mouse IgG isotype Control	Thermo Fisher Scientific	Cat#10400C
Calf Bovine Serum	ATCC	Cat#30-2030
Oil Red O	Sigma	Cat#O0625-25G
pluriStrainer 10 µm	PluriSelect	Cat#SKU43-50010-50
FlowTubes	Thermo Fisher Scientific	Cat#1152A25
Corning Thermowell GOLD PCR tues	Corning	Cat#CLS3750-1000EA
Mormal Goat Serum (10%)	Thermo Fisher Scientific	Cat#50062Z
STEMdiff APEL2 Medium	StemCell Technologies	Cat#05270
Electron Micrscopy Sciences 16% PFA	Fisher Scientific	Cat#50-980-487
M-PER Mammalian Protein Extraction Reagent	Thermo Fisher Scientific	Cat#78501
10X PBS (Phosphate Buffered Saline), pH7.4	VWR	Cat#75800-994
